# Developing a theoretically informed education programme within the context of a complex implementation strategy in UK primary care: an exemplar from the IMP^2^ART trial

**DOI:** 10.1186/s13063-022-06147-6

**Published:** 2022-04-23

**Authors:** Kirstie McClatchey, Viv Marsh, Liz Steed, Steve Holmes, Stephanie J. C. Taylor, Sharon Wiener-Ogilvie, Julia Neal, Rhian Last, Ann Saxon, Hilary Pinnock, for the IMP2ART Programme Group

**Affiliations:** 1 Asthma UK Centre for Applied Research, Usher Institute, Old Medical School, The University of Edinburgh, Teviot Place, Edinburgh, EH8 9AG UK; 2 Institute for Population Health Sciences, Barts and the London School of Medicine and Dentistry, Queen Mary University of London, London, UK; 3 The Park Medical Practice, Shepton Mallet, Severn School of Primary Care, Health Education England (South West), Bristol, UK; 4 Healthcare Improvement Scotland, Edinburgh, UK; 5 NHS Herefordshire and Worcestershire Clinical Commissioning Group, Worcester, UK; 6 Royal College of General Practitioners Yorkshire Faculty, Cheshire, UK; 7 Education for Health, Warwick, UK

**Keywords:** Asthma, Self-management, Primary care education, Online education, Education development

## Abstract

**Background:**

IMPlementing IMProved Asthma self-management as RouTine (IMP^2^ART) is a programme of work developing and evaluating a strategy for implementing supported asthma self-management in UK primary care. The strategy encompasses patient-facing resources, professional education, and organisational approaches to embed supported self-management. This paper reports the development of a theoretically informed interprofessional education programme which aims to raise awareness of and enable healthcare professionals to deliver effective supported self-management.

**Methods:**

Aligned with the Medical Research Council (MRC) Complex Intervention Framework, the multidisciplinary team developed educational content in three phases: (1) developmental phase, identifying educational and behaviour change theory to guide development, in consultation with a professional advisory group; (2) feasibility pilot phase, testing the education using a ‘think-aloud’ method; and (3) pre-pilot phase, delivering the education within the IMP^2^ART strategy.

**Results:**

The developmental phase identified educational and behaviour change theory and the need to provide two education modules: (1) a team module to raise awareness of supported asthma self-management for the whole team and (2) an individual study module for those who conduct asthma reviews with patients. The feasibility pilot highlighted content and design features in need of refinement and the pre-pilot identified substantial changes to the delivery strategy for the education modules.

**Conclusions:**

A multi-stage development process, aligned with the MRC Framework, contributed to the module design and delivery. Prior explorative work, multi-disciplinary team discussions, and professional advisory group consultation, informed the initial development, and in-practice testing and pre-pilot stages enabled refinement. In our experience, there were important benefits of working together as an educationalist/researcher team. The education programme, a core component of the implementation strategy, is now being tested in the IMP^2^ART UK-wide cluster randomised controlled trial.

## Background

Asthma is a long-term condition that affects approximately 262 million people globally [1] and 5.4 million people UK-wide [2]. Supported self-management for asthma, encompassing patient education, regular clinical review, and personalised asthma action plan provision, has been recommended by national and global asthma guidelines for 30 years [3, 4]. A recent meta-review with studies from at least 29 countries found that supported asthma self-management reduces hospitalisations, accident and emergency attendances, and unscheduled care [5]. Despite this, supported self-management, an evidence-based complex intervention, is poorly implemented in clinical practice. A recent Asthma UK survey found that less than half of respondents used an asthma action plan [6]. Furthermore, the UK National Review of Asthma Deaths highlighted that only 23% of those who died from asthma had an action plan [7].

Implementation of complex interventions, such as supported self-management [8], requires an organisational approach in addition to strategies directed at both staff and patients [9], and thus an approach to learning that considers the whole interprofessional team as important. The World Health Organization (WHO) defines interprofessional education as “students from two or more professions learn about, from, and with each other to enable effective collaboration and improve health outcomes” [10]. This educational approach improves collaboration and team working [11–13], as it supports individuals to develop a better understanding of roles, abilities, and responsibilities of others in the team. Aligned with the proposed reforms of the Lancet Commission on global health professional education [14], the WHO framework for Interprofessional Education and Collaborative Practice places interprofessional education at the heart of effective collaborative practice, strengthening healthcare systems and leading to improved health outcomes for patients [10].

IMPlementing IMProved Asthma self-management as RouTine (IMP^2^ART) is a programme of work developing and testing, in a cluster randomised controlled trial, a strategy for implementing supported asthma self-management in UK primary care [https://www.ed.ac.uk/usher/imp2art]. Along with patient-facing and organisational resources, team-based professional education is a core component of the strategy that seeks to embed supported self-management within practice routines. This paper reports the development, within the context of the IMP^2^ART research, of a theoretically informed interprofessional education programme which aims to raise awareness of and enable healthcare professionals to deliver effective supported self-management.

## Methods

Our programme of work aligns with the developmental and feasibility piloting stages of the Medical Research Council (MRC) Framework for developing and evaluating complex interventions [15] and follows the guidance for reporting intervention development studies in health research (GUIDED) [16]. The education is set within the multi-theories model of adult learning proposed by Taylor and Hamdy [17], which provides a framework for utilising and combining a range of educational principles and approaches. Ethical approval was provided by West Midlands - Black Country Research Ethics Committee (REC ref: 18/WM/0300), and all participants provided written informed consent.

### The multidisciplinary team

The IMP^2^ART education programme was developed by a multidisciplinary team who, in addition to e-mail correspondence, held a series of eight workshop/meetings from September 2018 to August 2019 both virtually and in-person.

#### Asthma UK Centre for Applied Research: IMP^2^ART team

The IMP^2^ART research team consisted of academics, general practitioners, and health psychologists based within the Asthma UK Centre for Applied Research (AUKCAR). Funded by Asthma UK, AUKCAR is a network of researchers from universities across the UK, people affected by asthma, healthcare professionals, NHS partners, and other organisations.

#### Education for Health

Education for Health (EfH) is a health education charity that aims to improve the lives of people living with long-term conditions. The EfH team included educationalists and clinical educators with expertise in curriculum design for adult learners, as well as online learning technologists with expertise in instructional design.

#### Professional advisory group

We established a Professional Advisory Group (*n* = 10) including doctors and nurses from the Primary Care Respiratory Society (PCRS) to advise on educational content. They met formally (by video-conference) on two occasions during the education development, with additional informal input from some members. Specific topics discussed included insights into the primary care context, practical barriers to implementing supported self-management and strategies for overcoming challenges.

### MRC framework

**Fig. 1:**
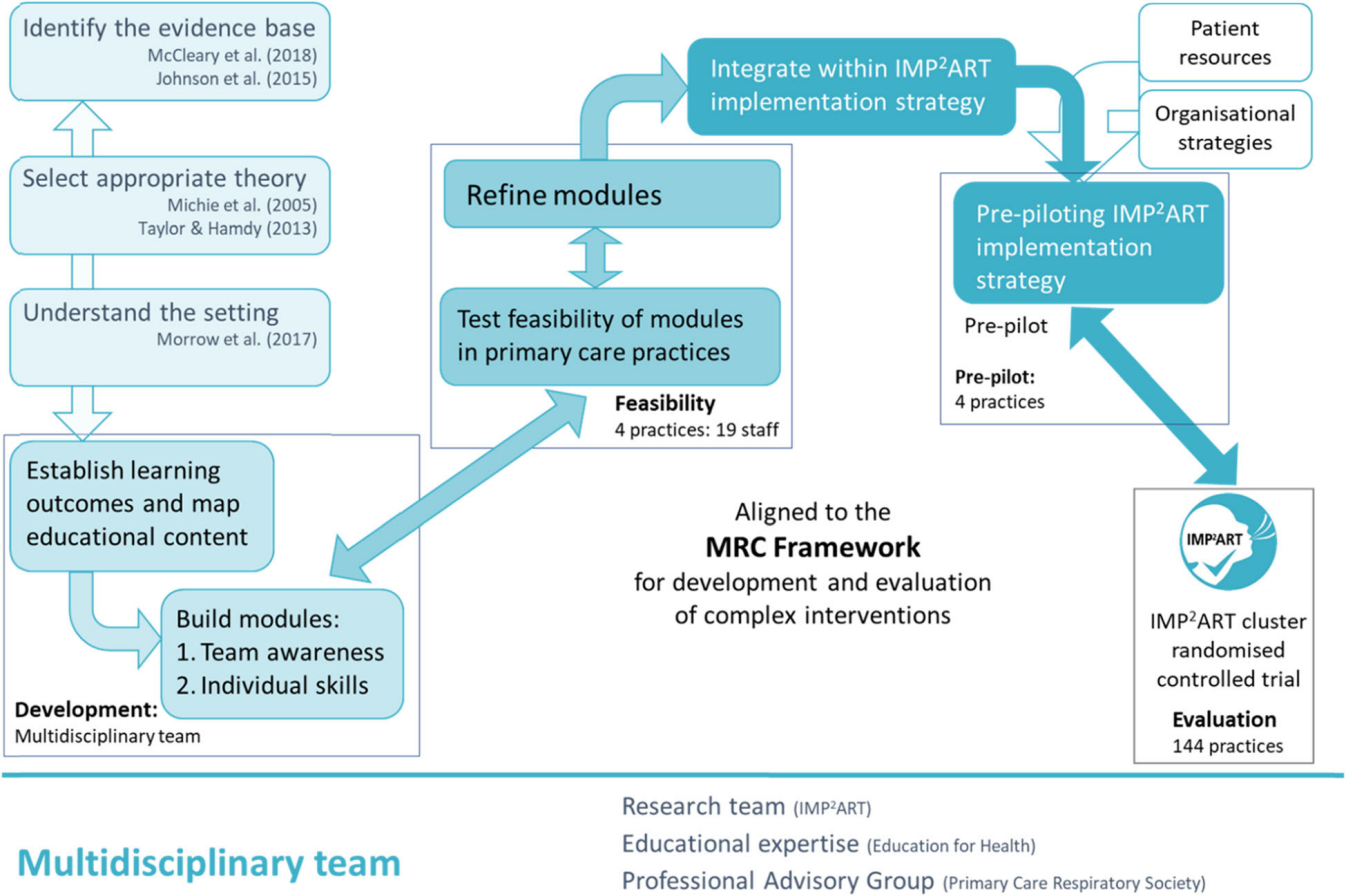
Phases of the IMP^2^ART education programme development

The phases of the education development mapped to the MRC Framework are illustrated in Fig. 1.

#### Developmental phase

Preliminary work included a systematic review [9], qualitative exploration of the primary care context [18], and an understanding of both the educational and behaviour change literature [19]. The developmental work was led by EfH (VM) in partnership with IMP^2^ART researchers (KM, LS, and HP) with advice from the Professional Advisory Group (led by SH). The learning aims and outcomes for the education were developed using existing theory, e.g. multi-theories model of adult learning [17], Bloom’s taxonomy [20], Theoretical Domains Framework [21], and the existing evidence-base [9, 18, 19].

#### Feasibility pilot phases

We recruited staff from four demographically diverse general practices from around the UK and delivered prototype versions of the education. We used a ‘think-aloud’ method [22], in which general practice staff were encouraged to verbalise their thoughts as they worked through the programme. A brief follow-up interview or focus group used a topic guide developed in-line with guidance by Creswell and Creswell [23], to clarify participants’ thinking about whether the aims and learning outcomes of the education programme were met, whether theoretical elements were addressed, and explored potential implementation. The think-aloud method was used to capture any changes that needed to be made to (for example) user friendliness or content of the education. Think-aloud sessions and follow-up interview/focus groups were carried out by an IMP^2^ART researcher (KM), were audio-recorded following consent, and transcribed verbatim. Time to complete the education was not recorded, as vocalising thoughts within the think-aloud methodology would have distorted findings. The sessions were analysed using thematic analysis [24], and all analyses were performed in NVivo 11.

#### Pre-piloting the education programme within the IMP^2^ART implementation strategy

Following necessary refinements, the educational components were integrated with the patient resources and organisational components of the IMP^2^ART implementation strategy for pre-piloting in an additional four general practices. Following the delivery of the pre-pilot, a sample of general practice staff from each of the four pre-pilot practices were interviewed by KM between October and November 2019. The semi-structured interviews followed a topic guide that aimed to explore staff experiences of the programme. Interviews were audio-recorded, transcribed verbatim, and analysed using thematic analysis [24] in NVivo 11.

## Results

### Developmental phase

#### Identify the evidence base

Our systematic review of education for healthcare professionals implementing supported asthma self-management informed the IMP^2^ART strategy [19]. Although findings were mixed, there was evidence to suggest that professional education, especially team-based education, can increase asthma action plan provision, improve asthma control, and reduce unscheduled care. Effective interventions were explicitly guideline-based, often involved local opinion leaders, and included interprofessional education. This echoes wider literature that suggests that educational outreach can change professional behaviour and improve processes of care by modifying peer group norms and expectations [25].

#### Selecting appropriate theory

Utilising well-established educational theory enabled us to understand our learners and to plan approaches to meet our goals and their learning needs. From the outset, we applied the overarching principles of andragogy (adult learning), which considers that motivation to learn is intrinsic and education should be learner-centred [26]. The curriculum design was grounded in cognitivist and constructivist theory aiming to build upon existing knowledge and develop deeper levels of understanding to enable application to practice. Bloom’s original taxonomy [20] supported the development of learning outcomes appropriate to the learner context. Taylor and Hamdy’s [17] multi-theories model provided a framework for exploring learning “phases” and drawing upon multiple theories to meet learning needs. Online module design was informed by learning style theory [27, 28] and transactional distance theory [29]. For example, visual, auditory, read/write, kinaesthetic (VARK) [27] principles allowed the full range of learning styles to be addressed via a variety of tasks, activities, interactions and narratives, and transactional distance was mitigated by interactive module design and scaffolding provided by additional resources and facilitator support.

Implementing supported self-management involves a change in professional behaviour [9]. Throughout the IMP^2^ART programme, the ‘COM-B’ framework of understanding behaviour was applied [30], with specific theoretical elements needed in an education programme identified and addressed through the Theoretical Domains Framework (TDF) [21]. The TDF describes and aids application of theory to the understanding and development of interventions to promote behaviour change, including that of healthcare professionals [31, 32]. The IMP^2^ART systematic review identified that the TDF domains ‘social influences’, ‘environmental context and resources’, ‘behavioural regulation’, ‘beliefs about consequences’, and ‘social/professional role and identity’, were associated with effective asthma education for healthcare professionals [19], and were therefore priorities for inclusion in the IMP^2^ART education.

#### Understanding the setting

We used findings from our prior qualitative study to inform the educational development, which explored how asthma care was delivered in UK general practice, and the views of clinicians and support staff on the skills they needed to support asthma self-management [18]. For practices in England, an important context is the Quality and Outcome Framework which rewards provision of an action plan [33], though there are concerns that this represents a ‘tick box’ rather than meaningful engagement with supported self-management [34, 35].

Supporting self-management was described as ‘high priority’ (particularly by nurses) but was viewed as a nurse-led role, with general practitioners (GPs) often feeling unfamiliar with the practicalities of providing asthma action plans. Improving practitioner skills and confidence was thus a priority, and this needed to include GPs, nurses, and administrative staff. Linking with the COM-B element of ‘social opportunity’ [30], we decided that the IMP^2^ART education programme should target the whole general practice team, and include two modules with the aim of:

1. Addressing the importance of supported self-management in asthma—for the whole practice team (team-awareness module: module 1),
2. Providing an in-depth education module on supporting self-management for the healthcare professionals who conduct annual asthma reviews with patients (most often nurses in the UK) (individual study module: module 2).

The Professional Advisory Group highlighted the need to reiterate evidence that supported self-management reduces asthma attacks and deaths and to discuss team roles within the practice (including the receptionist/administrative role in supporting self-management). There was concern that some healthcare professionals delivering asthma care may not have the recommended pre-requisite asthma training [36] and would not have the basic knowledge on which module 2 builds. This should thus be made explicit in the introduction to module 2 and suitable basic training signposted. In addition, the IMP^2^ART facilitators will be able to discuss training needs if they detect a lack of general asthma knowledge in the healthcare professional delivering care. The Professional Advisory Group also identified barriers that they had encountered in practice e.g. nurses lacking confidence in their ability to offer self-management advice, and patients viewing an action plan as ‘non-essential’. Their suggested solutions to overcoming these barriers included specific training on personalising action plans so the healthcare professionals felt confident to adapt/‘cross-out’ sections of the action plan if they were irrelevant for individual patients.

#### Establishing learning outcomes and mapping educational content

The aims and learning outcomes for each module were underpinned by the educational and behaviour theory and the evidence-base [9, 18, 19] and informed by discussions with the Professional Advisory Group (see Table 1 for details).

**Table 1:** Module 1 and 2 aims, learning outcomes and content

	Team-awareness module: module 1	Individual study module: module 2
Aim	To raise awareness of the benefits of supported self-management and increase engagement, motivation and commitment to supporting self-management (SSM) so that it becomes a priority across the whole practice team	To enable healthcare professionals to use behaviour change strategies in clinical practice to deliver effective supported self-management
Learning outcomes	▪ Understand the principles of supported self-management ▪ Recognise the benefits of supported self-management for patients and the practice ▪ Identify roles and contributions in the team approach to supported self-management	▪ Understand the concept of supported self-management ▪ Identify individual and organisational barriers to effective supported self-management ▪ Use a range of strategies to support behaviour change ▪ Reflect on various approaches to supported self-management ▪ Evaluate self-management resources appropriate for their patient population and context
Core module content	▪ Introduction (e.g. The National Review of Asthma Deaths (NRAD); definition of supported self-management) ▪ Principles of supported self-management (importance of SSM and barriers to SSM) ▪ Benefits of supported self-management (benefits for patients, general practice, and society) ▪ Teamwork (the team approach to supporting self-management) ▪ Summary, planning and evaluation	▪ Introduction (learners’ approach to and confidence in supporting self-management) ▪ What is supported self-management? (evidence for SSM) ▪ Barriers to effective supportive self-management (patient and healthcare professional perceptions, and overcoming barriers) ▪ Helping patients to change behaviour (behaviour change and communication skills) ▪ Approaches to supported self-management (working in partnership with patients and motivational interviewing) ▪ Self-management in practice (personalising asthma action plans) ▪ Summary, planning and evaluation ▪ Additional resources

Both modules 1 and 2 were developed with Nimble Author, an interactive eLearning development software [37]. As module 1 highlights a team-based approach to supporting asthma self-management, it was designed to be delivered by a facilitator in a whole-team setting (although it could also be completed individually online). The facilitator role development has been guided by the integrated Promoting Action on Research Implementation in Health Services (i-PARIHS) framework [38]. The module included 20 min of content to be delivered by the facilitator over an hour. Module 2, the education module for those who conduct asthma reviews with patients, was developed with 60 min of content.

The modules employed Taylor and Hamdy’s [17] five phases for learning. Learners existing knowledge is challenged in the *Dissonance* phase; solutions are found in the *Refinement* phase and solutions applied in the *Organisation* phase. Review and refinement occurs in the *Feedback* phase and learners reflect on action in the *Consolidation* phase. *Dissonance* was addressed in both modules by clarifying the task before learners engaged with it. Instructional design techniques such as reflective activities and team planning aligned to the *Refinement* and *Organisation* phases. Reflective activities have previously been identified as an important component for learning about self-management support [39]. *Feedback* was provided within the online modules, as responses to interactive learning activities such as quizzes and reflective activities. Problem-based learning within both modules provided opportunities for learners to *consolidate*, and this was enhanced by the facilitator who stimulated reflection on actions [40].

Module content was mapped to the TDF domains (including those associated with effective interventions in our systematic review [19]) to ensure all relevant behaviour change elements were included [41] (see Table 2 for details). The ‘social/professional role and identity’ domain was addressed by clearly defining potential roles of various staff/professionals in general practice. The ‘social influences’ domain was addressed by incorporating local opinion leaders’ views of supporting self-management (known to be associated with successful promotion of evidence-based practice [19, 43]). The domain ‘beliefs about consequences’ was addressed by providing information in the modules about health/societal costs of asthma and benefits of supported self-management. Other TDF domains, by nature relevant to professional healthcare education and training, such as ‘knowledge’ and ‘skills’, were also included in the education modules.

**Table 2:** Included module content related to relevant Theoretical Domains Framework (TDF) domains

Domains	Module 1 content	Module 2 content
*Knowledge*	Provision of evidence-base (e.g. The National Review of Asthma Deaths (NRAD) 2014 report; principles and importance of supported self-management (SSM)	Evidence and impact of SSM [8] Recommend clinical guidelines [42]
*Skills*	Building skills to work as a team to SSM	Consultation skills and spirit of motivational interviewing. How to co-create a personalised asthma action plan.
^a^*Social/professional role and identity*	Identifying role within their practice and team to SSM	Reflection exercise on ‘patients as partners’
*Beliefs about capabilities*	Reflection on whether module learning outcomes met	Measure of confidence in supporting patients to manage their asthma Reflection on whether module learning outcomes met
^a^*Beliefs about consequences*	Benefits of SSM to the patients; the general practice; the NHS; and to society	Benefits of SSM to the patients; the general practice; the NHS; and to society
*Reinforcement*	Provision of a certificate upon completion of the module.	Provision of a certificate upon completion of the module.
*Intentions*	Development of a 3–5 point team action plan at the end of the module of how the whole-practice team will support self-management in their practice	Identifying actions that can be utilised to support patients to self-manage their asthma
*Goals*	Development of a 3–5 point team action plan at the end of the module of how the whole-practice team will support self-management in their practice	Identifying actions that can be utilised to support patients to self-manage their asthma
^a^*Environmental context and resources*		Highlighting the available resources to provide SSM e.g. invitation letters for patients; asthma review templates that can be used in consultation; a range of asthma action plans
^a^*Social influences*	Interactive map with key local opinion leaders in their area discussing the importance of SSM Highlighting of whole-team approach to SSM	Highlighting team approach to SSM
*Emotion*	Provision of NRAD 2014 report findings	
^a^*Behavioural regulation*	Identifying barriers to SSM	Identifying barriers to effective SSM, and overcoming barriers in practice

^a^Identified by McCleary et al. [19] as having some evidence of effectiveness in educational initiatives for self-management support for asthma

Drawing on the VARK principals [27], both modules delivered content using a variety of methods to appeal to different learning styles e.g. traditional text, animated video, whiteboard animation (e.g. statistics on asthma deaths), and live-action video (e.g. patient stories recorded by the IMP^2^ART Patient and Public Involvement (PPI) team and key opinion leader views).

### Feasibility pilot phases

#### Learnings from the feasibility testing of the modules

A total of 19 general practice staff across four general practices participated in testing the modules. Seventeen practice staff (administrative staff (*n* = 8), nurses (*n* = 5), and GPs (*n* = 4)) participated in testing the whole-practice team-awareness module (module 1) facilitated in the practices (*n* = 2) by one of the authors (VM). Three nurses from three practices tested module 2, the individual study module (one of whom had also taken part in module 1 testing). We describe below the overarching themes derived from the modules 1 and 2 feasibility pilot data. An overview of the themes is displayed in Table 3 (module 1) and Table 4 (module 2).

**Table 3:** Module 1 themes

Overarching theme	Sub-theme	Illustrative quote
Delivery in a group setting	Group setting experience Considerations	“It’s probably the best way to get the maximum number of people aware at one point…” “…in a big group where you’ve got a huge range of staff, if the GPs start speaking, the admin staff will step back and not contribute.”
User experience	Clarity Animation and videos Design issues	“I think it certainly gets the information across.” “I think the more visual the better, in my experience, I like animation and videos.” (Observation; no illustrative quote)
Content	Raising awareness of SSM Relevance for all staff Examples of asthma action plans certification	“I think it’s really beneficial from a clinical point of view because it’s a reminder to look for the asthma action plan.” “I can imagine it would be interesting to non-medical staff probably more than medical staff. I didn’t hear anything that I didn’t know. But it wasn’t aimed at me solely so that’s fine.” “If it’s about a self-management plan, let’s have a look at one.” “…if you have, even if it’s one sheet, if you have some kind of certificate at the end…”

**Table 4:** Module 2 themes

Overarching theme	Sub-theme	Illustrative quote
User experience	Clarity Animation and videos Design issues Timing	“…easy to read. Not too much on the screen, the screen wasn’t too busy, which is always appreciated. Yeah, I thought the layout was fine, easy to read, flowed easily.” “The patients, yeah, seeing the videos of the patients. That always kind of helps to put it in perspective, I think. Yeah, I really liked that.” “…I’ve not actually got a speaker on here.” “…the module itself is going to take a lot longer for people to do and I think they do need time then to go… especially if you’re going to do a little bit of reflective things in it…”
Content	Positive aspects Behaviour change techniques Possible changes	“…the fact that you’ve got two very senior people, very experienced people, explaining it and talking it through should emphases to those nurses who don’t use it why they should.” “Actually, what I did get from it was it reminded me, just watching that flu vaccination interaction, it reminded me how theoretical motivational interviewing is and how in practice it’s much quicker.” “…the concentration was on the peak flow but the other bits I think could have been expanded on as well.”

Module 1:

- *Delivery in a group setting*. General practice staff were positive about module 1 being delivered to the whole team which they felt would raise awareness of supported self-management, though some clinicians were concerned that administrative staff may be less comfortable to contribute.
- *User experience*. The module was found to be clear. The included animations and videos were positively received by staff, particularly the videos with patients describing their experiences. There were a few practical issues, for example, sound tended to be disabled on practice computers, and some text inputted by the facilitator appeared in subsequent boxes.
- *Content*. The module raised awareness of supported self-management, though including an example of an asthma action plan was suggested. Clinicians appreciated some of the practical messages (e.g. ‘remember to look at the patients’ asthma action plan), though some felt that the content did not expand their existing knowledge. Staff suggested a certificate of completion would count towards their Continuing Professional Development (CPD).

Module 2

- *User experience*. Participating nurses found the module to be clear and easy to read, though there were some practical issues. The use of both animated and live video was welcomed, with the patient experience videos being well received. There were mixed opinions about the duration of the module, with some feeling that it would take about an hour, and others feeling it may take longer.
- *Content*. Positive aspects of the content included the use of credible sources in videos (e.g. two expert nurses describing the benefits and personalisation of action plans). Participants felt that the module would also be particularly useful for newer members of staff/GPs. The inclusion of behaviour change techniques to use with patients (e.g. motivational interviewing) was considered useful and a reminder for those already trained. One nurse suggested expanding on symptom information (e.g. coughing and expectorating).

### Refining the modules after feasibility testing

Following feasibility testing, a number of refinements were made. Module 1 was designed to be delivered by a facilitator, who needed to highlight the whole-team approach, and encourage all members of staff to participate, specifically addressing concerns that administrative staff in some practices may lack confidence to contribute to the session. Examples of asthma action plans will also be brought to facilitated module 1 sessions. In order to keep module 2 to time, instructions for exercises where the participant had to input information, e.g. reflection exercises, were revised to specify ‘brief notes’ or ‘three to five bullet points’. Further, some of the exercises were added to a separate ‘resources’ section to ensure that the learner could complete the module within 1 h. For both modules 1 and 2, design issues were fixed and, to circumvent the lack of sound on some general practice computers, a transcription was added to all videos. Finally, as an incentive to encourage completion of the modules, we added completion certificates, which learners could use towards their CPD.

### Findings related to the education modules within the IMP^2^ART implementation strategy pre-pilot

The pre-pilot provided practices with the refined education modules, along with other components of the IMP^2^ART implementation strategy including patient-facing resources (e.g. patient invitation letters, an asthma information website), organisational components (e.g. audit and feedback, a patient-centred asthma review template). Module 1 was delivered by a facilitator (VM or RL) in four pre-pilot general practices. Access to Module 2 was provided for the clinician(s) who conducted asthma reviews in each of the four practices. It became clear that a proactive approach was needed to ensure whole team attendance at the facilitated module 1 session; one practice had only invited core staff. Participants indicated that they would welcome the module link being available to them prior to the session “…giving it to us beforehand just to look through, you know.” It also became clear that the facilitator needed flexibility to adjust the session to the priorities of the individual practice e.g. to introduce a specific IMP^2^ART resource that the practice might wish to adopt. Finally, for module 1, the team task of developing a ‘practice plan’ to embed supported self-management needed greater emphasis. Completion of module 2 was low, and reminders about availability of this module were needed.

#### Refinements after pre-pilot

We developed a sequence of emails to be sent to all practice staff in the four weeks leading up to the facilitated module 1 session promoting the session and highlighting its applicability to all staff. The emails also provided a link to module 1, so that staff could (as an option) explore the material prior to the session. The approach to facilitation was changed so that module 1 could be delivered flexibly, tailoring the session to the individual practice, in order to improve motivation and engagement rather than “teaching”. After developing the ‘practice plan’ in the facilitated module 1 session, scheduled follow-up contact with the practice reminded staff about the plan to which they had agreed and encouraged them to work towards implementing supported asthma self-management in their practice. We developed reminder emails for clinicians who were eligible to complete module 2.

### Adaptation to COVID-19 context

In mid-2020 and post-refinements, we held a virtual workshop with IMP^2^ART educationalists and researchers to finalise the implementation strategy. UK primary care was adapting to remote consultations due to the COVID-19 pandemic [44], and we iteratively added content to module 2 that covered effective remote consultation skills. No content changes were made to module 1, as the flexible facilitation strategy meant the module could be delivered face-to-face (as originally intended) or remotely via video-conference. The final content of the two modules is summarised in Table 1.

## Discussion

In summary, we describe the development within a research programme of two asthma self-management online education modules. One facilitated module highlighted the importance of a team approach and one provided education for those who conduct asthma reviews with patients. Aligned to the MRC Framework [15], the process included a developmental phase drawing on and mapping to educational and behaviour change theories, a feasibility phase to test module content and design, and a pre-pilot of the education modules as components of the IMP^2^ART whole-systems implementation strategy. Throughout the iterative process, changes were made to both the educational content and design, as well increasing flexibility of the facilitation. The IMP^2^ART strategy (incorporating the education programme) is now being tested in an internal pilot and UK-wide cluster randomised controlled trial (ISRCTN registry, ref: ISRCTN15448074). If successful, this has potential to change the way that asthma care is delivered in primary care, by directing focus and highlighting the importance of supported self-management.

### Strengths and limitations

A major strength was the cross-discipline empirical and theoretical work underpinning development of the asthma self-management education. The modules were developed using a systematic and standardised approach with a multi-disciplinary team and used appropriate educational and behaviour change theory, existing evidence, and stakeholder advice. Feasibility testing, pre-piloting and refining allowed for necessary changes to be made to the education programme. In addition, the modules were developed and tested with current general practice staff, strengthening applicability to real-world practice. Our thorough description of the development and content of the modules avoids the criticism of a recent systematic review that the training for asthma educators should be better described [45] and may act as a model/framework for other primary care staff course development. A limitation was the poor initial uptake of module 2. Refinements to the delivery and strategies to facilitate engagement were made, though until the pilot trial is conducted, the impact of this strategy will not be clear. IMP^2^ART is a UK programme of work and would need to be adapted to other healthcare systems in which the professionals delivering asthma education and supporting self-management may be different. The principles of taking a multi-disciplinary approach to developing and adapting an education intervention are however, transferable.

### Interpretation of findings in relation to previously published work

Recent commentaries have suggested that medical education might benefit from working within a complex intervention framework [46, 47]. We used this approach and embedded the development, piloting and testing of educational modules within the MRC complex intervention framework [15]. Our multidisciplinary team worked together so that researchers learnt from educational theory [17, 20] and the educationalists gained by discussing behaviour change techniques with health psychologists [21] and implementation frameworks with researchers [48]. The educational modules benefitted by being conceived within a whole systems implementation strategy, which added an over-arching dimension and changed the delivery of one of the modules. A systematic overview that used Normalization Process Theory to analyse studies of complex interventions [25] concluded that educational meetings and outreach were more effective when ‘bundled together’ with strategies such as audit and feedback and practical organisational tools that modified the peer group norm and influenced structures of practice (a target of IMP^2^ART module 1). This comprehensive developmental process has potential implications for the development of future healthcare education seeking to change professional behaviour.

The MRC Framework imposed a structured process of feasibility testing and we built on this by incorporating a real-world pre-pilot as part of the intervention design, an approach taken by other complex interventions in primary care [49]. This iterative approach to development enabled us to refine the educational modules and adapt them further when the COVID-19 pandemic altered the primary care context. Furthermore, practices will be encouraged to adapt the components of the implementation strategy to their practice profile, the skill mix of their practice staff, and the routines of their practice organisation. Evaluation will assess implementation (action plan ownership) and health outcomes (unscheduled care) in a UK-wide cluster randomised trial (ISRCTN registry, ref: ISRCTN15448074). This corresponds to assessing changed behaviour and benefit to society described in (for example) Kirkpatrick’s hierarchy of levels of evaluation [50].

## Conclusion

We conclude that a multi-stage development process aligned with the MRC complex intervention framework contributed to the design and delivery of the education modules. Prior explorative work, multi-disciplinary team discussions, professional advisory group consultation, informed the initial development, and in-practice testing and pre-pilot stages enabled refinement. Not all education can/should be part of a whole systems implementation strategy, and evaluating outcomes at the level of changing practice and improving health outcomes is not always feasible. There are, however, lessons to be learnt from our experience of working together in an educationist/researcher team.

## Data Availability

The data that support the findings of this study are available from the corresponding author upon reasonable request.
